# Apnea test for brain death diagnosis in adults on extracorporeal membrane oxygenation: a review

**DOI:** 10.5935/0103-507X.20200048

**Published:** 2020

**Authors:** Erica Regina Ribeiro Sady, Lígia Junqueira, Viviane Cordeiro Veiga, Salomón Soriano Ordinola Rojas

**Affiliations:** 1 Unidade de Terapia Intensiva Neurológica, Hospital Beneficência Portuguesa - São Paulo (SP), Brasil.

**Keywords:** Extracorporeal membrane oxygenation, Brain death/diagnosis, Point-of-care testing, Intensive care units, Oxigenação por membrana extracorpórea, Morte encefálica/diagnóstico, Testes imediatos, Unidades de terapia intensiva

## Abstract

Among the potential complications of extracorporeal membrane oxygenation, neurological dysfunctions, including brain death, are not negligible. In Brazil, the diagnostic process of brain death is regulated by Federal Council of Medicine resolution 2,173 of 2017. Diagnostic tests for brain death include the apnea test, which assesses the presence of a ventilatory response to hypercapnic stimulus. However, gas exchange, including carbon dioxide removal, is maintained under extracorporeal membrane oxygenation, making the test challenging. In addition to the fact that the aforementioned resolution does not consider the specificities of the diagnostic process under extracorporeal membrane oxygenation, studies on the subject are scarce. This review aims to identify case studies (and/or case series) published in the PubMed^®^ and Cochrane databases describing the process of brain death diagnosis. A total of 17 publications (2011 - 2019) were identified. The practical strategies described were to provide pretest supplemental oxygenation via mechanical ventilation and extracorporeal membrane oxygenation (fraction of inspired oxygen = 1.0) and, at the beginning of the test, titrate the sweep flow (0.5 - 1.0L/minute) to minimize carbon dioxide removal. It is also recommended to increase blood flow and/or sweep flow in the presence of hypoxemia and/or hypotension, which may be combined with fluid infusion and/or the escalation of inotropic/vasoactive drugs. If the partial pressure of carbon dioxide threshold is not reached, repeating the test under supplementation of carbon dioxide exogenous to the circuit is an alternative. Last, in cases of venoarterial extracorporeal membrane oxygenation, to measure gas variation and exclude differential hypoxia, blood samples of the native and extracorporeal (post-oxygenator) circulations are recommended.

## Abstract (pt)

Entre as potenciais complicações da oxigenação por membrana extracorpórea, as disfunções neurológicas, incluindo morte encefálica, não são desprezíveis. No Brasil, o processo diagnóstico é regulamentado pela resolução 2.173 de 2017 do Conselho Federal de Medicina. Entre os testes diagnósticos, está o de apneia, que objetiva verificar se existe resposta ventilatória ao estímulo hipercápnico. Contudo, trocas gasosas, incluindo a remoção de dióxido de carbono, são mantidas sob oxigenação por membrana extracorpórea, tornando o teste desafiador. Somado ao fato de que a citada resolução não contempla as especificidades do processo diagnóstico sob oxigenação por membrana extracorpórea, publicações sobre o tema são escassas. Esta revisão objetivou identificar estudos de casos (e/ou séries de casos) publicados nas bases PubMed^®^ e Cochrane que descrevessem o processo. Foram identificadas 17 publicações (2011 - 2019). As estratégias práticas descritas foram: prover oxigenação suplementar pré-teste, via ventilador mecânico e oxigenação por membrana extracorpórea (fração inspirada de oxigênio = 1,0), e, ao início do teste, titular o *sweep flow* (0,5 - 1,0L/minuto), a fim de minimizar a remoção de dióxido de carbono. Recomenda-se também incrementar o fluxo sanguíneo e/ou do sweep ante hipoxemia e/ou hipotensão, podendo associar à infusão de fluidos e/ou ao escalonamento de drogas inotrópicas/vasoativas. Se o limiar da pressão parcial de dióxido de carbono não for alcançado, repetir o teste sob suplementação de dióxido de carbono exógeno ao circuito é uma alternativa. Finalmente, nos casos de oxigenação por membrana extracorpórea venoarterial, para mensurar a variação de gases e excluir hipóxia diferencial, recomenda-se coletar amostras sanguíneas provenientes das circulações nativa e extracorpórea (pós-oxigenador).

## INTRODUCTION

Extracorporeal membrane oxygenation (ECMO) is a life support therapy aimed at assisting cardiac and/or respiratory function.^(1)^ Its role is established in more severe cases and is refractory to conventional therapies, which, however, must be potentially reversible.^(1,2)^

According to the Extracorporeal Life Support Organization (ELSO) recommendations for adults, ECMO for respiratory support should be considered in cases of acute hypoxemic respiratory failure, such as in acute respiratory distress syndrome; carbon dioxide retention on mechanical ventilation (MV) despite high plateau pressure; severe air leak syndromes; and/or the need for intubation while awaiting lung transplantation.^(3)^

For cardiovascular support, the indications are refractory cardiogenic shock, evidenced by inadequate tissue perfusion, secondary to hypotension and low cardiac output despite adequate intravascular volume, administration of fluids, inotropes and/or vasoconstrictors, and intra-aortic balloon assistance, when appropriate.^(4)^ In addition, ECMO is also indicated in cases of immediate cardiorespiratory collapse and, in some situations, septic shock.^(4)^

Among the potential complications, the incidence of neurological dysfunction is not negligible. Considering the tendency toward underestimation due to diagnostic limitations, it is estimated that 7 - 50% of patients on ECMO present with multiple conditions, including brain death (BD).^(5,6)^ Data show that the latter occurs in 21 - 28% of patients on ECMO with neurological complications.^(5-7)^

In Brazil, the diagnostic process of BD is regulated by the Federal Council of Medicine (*Conselho Federal de Medicina* - CFM) through resolution 2,173 of November 23, 2017.^(8)^ Diagnostic tests for BD include the apnea test, which aims to determine the absence of respiratory activity in the presence of hypercapnia.^(8)^ However, under ECMO, carbon dioxide removal is maintained by the oxygenator membrane, despite the absence of respiratory activity.^(9)^ Therefore, adjusting the device parameters is necessary so that the test can provide confirmation.

To understand the practical aspects of performing the apnea test for the diagnosis of BD in adult patients on ECMO, the objective of this study was to conduct a literature review on the subject.

## METHODS

**Figure 1:**
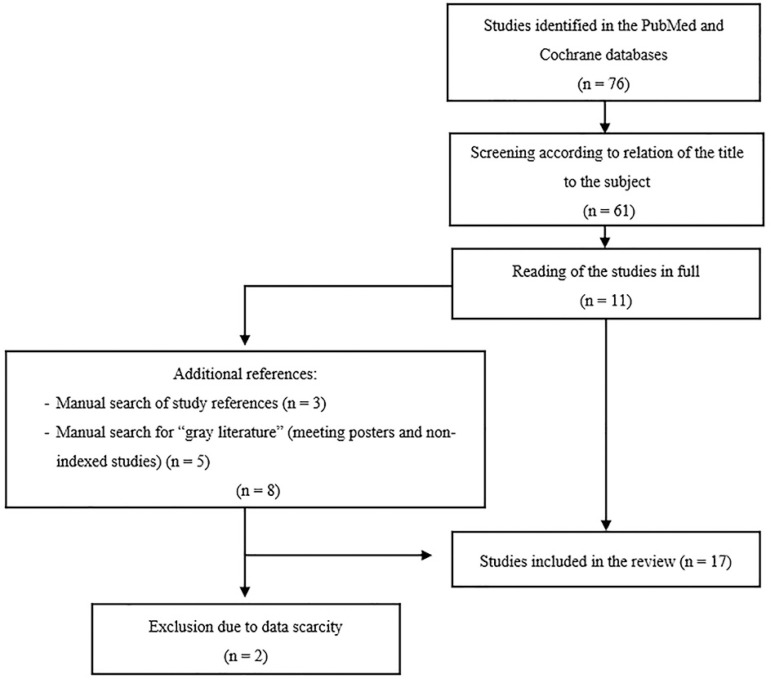
Flowchart of the study search process.

The PubMed^®^ and Cochrane databases were searched using the following keywords and operators: ((“brain death”) OR (“apnea test”) AND (“extracorporeal membrane oxygenation” or “ECMO”)). There were no restrictions as to the publication date of the studies. The inclusion criteria were case studies or case series with adult patients on ECMO, in any modality, with suspected diagnosis of BD, that described the execution of the apnea test. After identifying the subject by the title, the abstracts were read, and inclusion was confirmed after the full text was read. Additionally, the references cited in those studies were researched and included if the previously described inclusion criteria were met. Studies not indexed by the cited databases, including meeting posters, were also included as long as they met the inclusion criteria. Figure 1 illustrates the study inclusion process.

## RESULTS

A total of 76 studies were identified from the database search using the specified keywords. Among these studies, 61 were selected after reading the title. After reading the full text, 11 were selected. Additionally, eight studies were identified in the references of primary studies and through a manual search of publications not indexed by the cited databases, including meeting posters. Of these, two were excluded because they did not describe the execution of the apnea test. Thus, this review consists of 17 case studies or case series (67 individuals) published between 2011 and 2019 that described the apnea test in adults patients on ECMO.

## DISCUSSION

### Practical aspects of the apnea test under extracorporeal membrane oxygenation

The complexity of performing the apnea test is considered to be greater in situations of extracorporeal life support, which can be attributed to multiple factors, such as rarity of the procedure; reduced practical experience of the evaluators; and physiological instability resulting from the condition of BD, added to the severity of the condition that motivated the institution of ECMO as well as the presence of multimorbidities - common in critically ill patients.

An additional noteworthy factor is the interaction between the pro-inflammatory effects of exposure to the synthetic surfaces of the ECMO device that, in an environment of acidemia, such as that generated during the apnea test, predisposes to instability.

However, only five studies reported that the tests were abandoned, one motivated by the detection of respiratory effort after hypercapnic stimulus and the others by clinical instability.^(10-14)^ Hemodynamic instability was described by only three studies.^(10-12)^ Of these, only one was abandoned due to refractoriness to rescue interventions.^(12)^

Thus, in cases of hemodynamic instability, considering that in venoarterial ECMO there is presumed cardiac dysfunction, management aims to increase extracorporeal support by escalating the ECMO blood flow, combined or not with fluid infusion and/or escalation of inotropes.^(10)^ In turn, in venovenous ECMO, in which the probable cause of hypotension may be related to severe hypoxemia, in addition to providing supplemental oxygen therapy, alveolar derecruitment should be prevented using a positive end-expiratory pressure (PEEP) valve, i.e., continuous positive airway pressure (CPAP), external to or in the ventilator.^(9)^

In addition, it is possible to combine, if necessary, escalation of ECMO blood flow, of vasoactive drugs and, last, of sweep flow. The latter, however, requires attention because it can increase carbon dioxide removal. Fluids infusion is also suggested.^(9)^ However, in a scenario of underlying lung injury, we believe that this strategy should be used with caution.

In the absence of hemodynamic instability, as observed in the reviewed studies, the blood flow value should be kept fixed.^(2,12-24)^ In cases of instability refractory to the recommended adjustments, the test should be abandoned.

Hypoxemia is also a potential complication. Although more frequent than arterial hypotension, it was described by only four studies.^(10,12,13,23)^

Some advocate that oxygen supplementation during the test is not necessary for all patients on ECMO because adequate gas exchange is ensured if the device is programmed to provide blood flow at 75 - 80% of cardiac output - a management decision described by two studies.^(2,18)^ However, most suggest that supplementary oxygen therapy should be provided to maintain potential donor stability. Therefore, the consensus recommendation is to perform adequate preoxygenation for approximately 10 minutes with fraction of inspired oxygen (MV) = 1.0 (100%) and fraction of supplied oxygen (ECMO) = 1.0 (100%).^(2,12-25)^

Disconnection from the ventilator was performed in most of the reviewed cases, combined with supplemental oxygen therapy via catheter or T-piece.^(10,12-14,16,17,22,23)^ The use of PEEP was also frequent. In these cases, an external valve attached to the endotracheal tube was instituted via a T-piece (CPAP 5 - 10cmH_2_O) or attached to the resuscitation bag (bag-valve or AMBU^®^ bag). In addition, in one case, PEEP was supplied in the ventilator itself. ^(10,12,16)^

Additionally, in cases of significant respiratory dysfunction or to maintain lung function intact for transplantation, studies made reference to Giani et al., who proposed performing alveolar recruitment maneuvers pre- and posttest as an intervention protocol.^(10)^

In addition to the aforementioned strategies, escalating blood flow (ECMO) and/or careful re-escalating of sweep flow can be used in cases in which flow was reduced to very low values (< 0.5L/minute).^(12)^ In refractory situations, the test should be abandoned.

In cases of venoarterial ECMO, Ihle et al. warned of the fact that brain tissue may be exposed to differential hypoxia, which may not be detected by arterial blood gas analysis when the blood sample is derived only from the native circulation, for example.^(12)^ This occurs in cases in which the shunt point between it and the extracorporeal circulation occurs at the most distal site from the aorta.^(12)^

In this situation, the right cerebral hemisphere (or both brain hemispheres, depending on the location of the shunt point) would be perfused by anterograde blood flow from the pulmonary circulation.^(12)^ In the case of preserved cardiac function associated with significant respiratory dysfunction, these regions would be exposed to hypoxia, while the left cerebral hemisphere would be exposed to normoxic conditions because it would be perfused with retrograde blood flow from ECMO.^(12)^

However, because the exact determination of this shunt point is difficult, the authors consider it mandatory to collect blood samples from the circuit after the ECMO oxygenator, in addition to a sample from the arterial bed in the most peripheral path (right radial artery for femoro-femoral cannulation or femoral artery for axillary cannulation).^(12)^ For this reason, in a recent study, the authors recommend a target oxygen saturation of > 88% in the two samples, ensuring that both hemispheres are not exposed to differential hypoxia.^(12)^

In addition to the complications described, management related to carbon dioxide removal by ECMO is noteworthy, given that the absence of respiratory movements in the presence of hypercapnia is assumed to be compatible with the diagnosis of BD.^(8)^

If ECMO blood flow is kept constant, the concentration of carbon dioxide varies inversely, although not in a direct proportion, with sweep flow.^(9,15)^ Therefore, if adjustments to this parameter are not performed, it is not possible to evidence hypercapnia above the threshold required for validation of the apnea test.

Thus, although two studies described interrupted sweep flow, there is a tendency to recommend that at the beginning of the apnea test, the sweep flow should be reduced.^(16,17)^ The most frequently used values were 0.5 - 1.0L/minute.^(2,10,12,13,15,18-23-25)^ In addition, most studies recommend against reducing the sweep flow to below 0.5L/min, as this may predispose patients to hypoxemia and derail the completion of the test.

Even when following the abovementioned recommendation, hypercapnia above the threshold required by different legislations may not be achieved. In addition, cases in which the evaluator chooses not to reduce the sweep flow to prevent secondary hypoxemia should be considered.

In these cases, the solution proposed by Pirat et al.,^(19)^ Champigneulle et al.^(11)^ and Beam et al.^(2)^ is to supply carbon dioxide exogenous to the ECMO circuit via an adapter placed between the gas flowmeter/blender and the oxygenator. The procedure is relatively simple but requires the administration of carbon dioxide at a flow rate equivalent to the estimated rate of blood gas removal by the sweep gas.

Thus, in both strategies, continuous monitoring of the carbon dioxide concentration via capnometry is recommended, as should be done via pulse oximetry to maintain adequate oxygen saturation.^(12)^ For this purpose, frequent arterial blood gas samples can be collected at the discretion of the evaluator.^(12)^

Finally, in cases of venoarterial ECMO, in addition to preventing regional hypoxia, it should be ensured that the brainstem, in fact, is exposed to the hypercapnia stimulus.^(12,26)^ For that, Ihle et al. recommend measuring the blood gas tension of the native circulation, in the peripheral arterial pathway, and of the extracorporeal circulation by collecting samples in the ECMO circuit after the oxygenator.^(12)^ These authors also describe a case whose test was invalidated because the partial pressure of carbon dioxide (PaCO_2_) of the blood sample collected after the ECMO oxygenator was lower than the threshold required by legislation (60mmHg), despite the tension of the sample obtained from the native circulation being higher than the required threshold.^(12)^

Therefore, a PaCO_2_ above the threshold in both samples would be unequivocal proof that both brain hemispheres were exposed to this gas concentration and that, therefore, in the absence of respiratory movement (apnea), the test would be irrefutably compatible with the condition of BD.^(12)^

This strategy of dynamic and continuous assessment of gas tension in the native and extracorporeal circulations, with real-time parameter adjustments, although new, seems promising to assist professionals in the safe execution of the apnea test.

**Figure 2:**
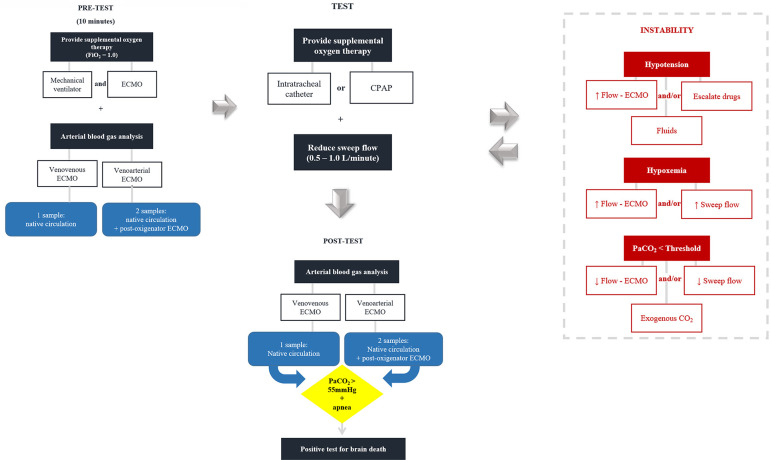
Strategies for the management of extracorporeal membrane oxygenation during the apnea test for the diagnosis of brain death. FiO_2_ - fraction of inspired oxygen; ECMO - extracorporeal membrane oxygenation; CPAP - continuous positive airway pressure; PaCO_2_ - partial pressure of carbon dioxide; CO_2_ - carbon dioxide.

Figure 2 shows the strategies for ECMO management during the apnea test.

### Ethical implications

A Brazilian study that conducted an analysis of the economic effect of the use of ECMO in the country suggested that its costs may be acceptable.^(27)^ However, the authors acknowledge that costs associated with the management of other organ dysfunctions, such as neurological complications, were not considered in the analysis.^(27)^

Therefore, efforts to properly perform the diagnostic assessment of BD in patients on ECMO are, in addition to being clinically necessary, ethical. This is because it allows, among other aspects, the rational and fair allocation of resources in intensive care units by detecting situations in which the maintenance of therapies is futile, as in cases of support to individuals with BD who are not candidates for organ donation. In this sense, the Federal Medical Council, through resolution 1,826/2007, notes “the legality and ethical character of suspending therapeutic support procedures when the BD of a non-organ donor patient is determined”.^(28)^

## FINAL CONSIDERATIONS

This review showed that despite practical challenges, the execution of the apnea test in adult patients on ECMO with clinical suspicion of BD is feasible. Management mainly includes but is not limited to adjusting the sweep flow and, in cases of instability, titrating the blood flow. In addition, counterintuitively, it was found that reports of complications were low.

This review discussed practical strategies that should be considered by health professionals and that, in the future, may contribute to the development of national technical recommendations, given that the use of extracorporeal membrane oxygenation, including in developing countries such as Brazil, is a reality.
